# South Tyrol (Italy) *Pastinaca sativa* L. subsp. *sativa* Essential Oil: GC-MS Composition, Antimicrobial, Anti-Biofilm, and Antioxidant Properties

**DOI:** 10.3390/molecules30143033

**Published:** 2025-07-19

**Authors:** Daniela Di Girolamo, Natale Badalamenti, Giusy Castagliuolo, Vincenzo Ilardi, Mario Varcamonti, Maurizio Bruno, Anna Zanfardino

**Affiliations:** 1 Department of Biology, University of Naples Federico II, 80126 Naples, Italy; daniela.digirolamo@unina.it (D.D.G.); giusy.castagliuolo@unina.it (G.C.); varcamon@unina.it (M.V.); anna.zanfardino@unina.it (A.Z.); 2 Department of Biological, Chemical and Pharmaceutical Sciences and Technologies (STEBICEF), University of Palermo, Viale delle Scienze, 90128 Palermo, Italy; natale.badalamenti@unipa.it (N.B.); vincenzo.ilardi@unipa.it (V.I.); 3 NBFC—National Biodiversity Future Center, 90133 Palermo, Italy

**Keywords:** *Pastinaca sativa* subsp. *sativa*, Apiaceae, octyl acetate, octyl butanoate, natural antimicrobial, antibiofilm property, antioxidant activity

## Abstract

*Pastinaca* L. is a small genus belonging to the Apiaceae family, traditionally used for both nutritional and medicinal purposes. *Pastinaca sativa* L. subsp. *sativa* is a biennial plant widely distributed in Europe and Asia, with recognized ethnopharmacological relevance. In this study, the essential oil (EO) obtained from the aerial parts of *P. sativa* subsp. *sativa*, collected in Alto Adige (Italy)—a previously unstudied accession—was analyzed by GC-MS, and the volatile profile has been compared with that of EOs previously studied in Bulgaria and Serbia. The EO was found to be rich in octyl acetate (38.7%) and octyl butanoate (26.7%), confirming that this species biosynthesizes these natural esters. The EO and its main constituents were tested to evaluate their antimicrobial properties. Furthermore, their biological potential was evaluated through antimicrobial, antibiofilm and antioxidant assays. This research work, in addition to evaluating possible chemotaxonomic differences at the geographical level of EOs of *Pastinaca sativa* subsp. *sativa*, has been extended to the determination of the biological properties of this accession never investigated before, with the aim of acquiring a broader vision of biofilm and antibacterial properties.

## 1. Introduction

The genus *Pastinaca* L. belongs to the Apiaceae family, including sixteen accepted species that grow from Europe to Mongolia and the Middle East [1]. The word *Pastinaca* could originate from the Latin word *pastus*, that can be translated as “food” [2].

Some *Pastinaca* species are utilized as medicinal plants due to their curative properties. For example, in Turkey, the husked and crushed roots of *Pastinaca sativa* subsp. *urens* Čelak. are externally used for wound healing and stomach-ache [3], or the infusion of *P. armena* Fisch. & Mey is used as an anti-inflammatory agent [4].

By far, the most popular medicinal plant is *P. sativa* L. (Parsnip). A tea made from its roots has been utilized in the treatment of women’s complaints, as diuretic, and for intermittent fever, whereas a poultice of the roots has been employed to treat psoriasis, and vitiligo cutaneous inflammations [5,6]. It is also recommended to treat dermatitis, stomatitis, headaches, ophthalmitis, and fever [6,7,8], and as a decoction is considered useful for bruising caused by frostbite, whereas food consumption of pickled parsnip helps solve kidney problems [9,10].

*P. sativa* is a biennial plant, more or less pubescent, high up to 100 cm. The stems are hollow or solid, angled or terete. The basal leaves are usually simply pinnate, rarely two-pinnatisect, with secondary veins inconspicuous and segments acute or obtuse, crenate–dentate; the teeth have a cartilaginous mucro and petioles are slender, not spongy. The rays (5–20) are angled. The flowers of this plant are small and white in colour which form a lumpy structure after maturation. Fruit (5–7 mm) are broadly elliptical, wing 0.25–0.5 mm wide and vittae on the commissural face not reaching the ends of the fruit.

Four subspecies are present in Europe: subsp. *sylvestris* (Miller) Rouy & Camus, subsp. *urens* (Req. ex Godron) Celak., subsp. *divaricata* (Desf.) Rouy & Camus, endemic to Corsica and Sardinia, and subsp. *sativa*, object of the present study [11].

The native range of *P. sativa* subsp. *sativa* is West and Central Europe, growing primarily in the temperate biome, but now it has spread in Asia, America, and some African countries, being widely cultivated. It has colonized old, abandoned farms, railroads, street sides and unused lands [1,2].

*P. sativa sensu latu* (which includes all the subspecies), known by different conventional names in several languages, such as *zardak* and “wild carrot” in Persian, *parsnip* in English, *cujtive and panipainais* in French, *jazar* in Arabic, *kajer* in Indian and *pastinaca* in Italian, is largely utilized as food. In the past it was confused with carrot until, in the 18th century, Linneo provided a definitive classification for the two species, naming carrot as *Daucus carota* and parsnip as *Pastinaca sativa* [12,13]. Its roots, sweeter than carrots, are eaten baked and can also be used in soups, stews, salads, casseroles, pies, and puddings. The leaves and young shoots, cooked with other greens, are used as a vegetable or added to soups. The seeds are used as condiment with a taste like dill [14,15].

Based on the increasing interest in Apiaceae EOs and their potential pharmacological activities [16], this study aimed to investigate the chemical composition and biological properties of the EO extracted from the aerial parts of *Pastinaca sativa* subsp. *sativa* collected in South Tyrol (Italy), a natural accession that has not been previously studied. Specifically, we evaluated its antimicrobial, antibiofilm, and antioxidant activities, along with those of its two major constituents, octyl acetate and octyl butanoate.

## 2. Results and Discussion

### 2.1. Gas Chromatography and Mass Spectrometry (GC-MS) Analysis of the EO

The composition of the EO of *P. sativa* subsp. *sativa* (**PSS**) was analyzed by GC-MS (Table 1). Twenty-nine compounds, divided into six different chemical classes, were identified and classified according to linear retention indices (LRIs). In terms of compound classes, esters (77.3%) dominate, by far, the EO’s composition, with octyl acetate (38.7%), octyl butanoate (26.7%), and hexyl butanoate (7.9%) as the most abundant compounds. Among the alcohols (5.8%), only 1-octanol (5.5%) is present in significant quantity. On the other hand, hydrocarbons, both monoterpenic and sesquiterpenic, occurred in very moderate amounts (3.3% and 2.3%, respectively). I = The presence is noteworthy, among the other compound classes (4.8%), of a discrete quantity of myristicin (2.9%), and a small amount of *γ*-palmitolactone (0.6%).

As shown in Table 2, which reports the major constituents (>3%) of the EOs of all *Pastinaca* species and ssp. studied so far, no Italian taxa or accessions have been previously investigated. 

Table 2 indicates also that only three taxa have been studied: *P. hirsuta* Pančić., P. *sativa* L. subsp. *urens* (Req. ex Godron) Celak., and *P. sativa* L. ssp. *sativa*, since the name reported in some publications, *P. silvestris* Mill. [25,26], *P. sativa* ssp. *sativa* var. *hortensis* [27], and *P. sativa* L. ssp. *sylvestris* [Mill.] Rouy and Camus [28] must be considered, according to POWO [1], as synonymous with *P. sativa* L. ssp. *sativa*.

The composition of **PSS** is in good agreement with the other previously studied EOs. In fact, linear esters are the main constituents of the aerial parts of almost all accessions, although they vary in composition and percentage, and they can be considered a marker of the genus. Octyl acetate, the main metabolite of **PSS**, has been identified as the principal product only in the EOs from the flowers of *P. silvestris* Mill. (Syn: *P. sativa* ssp. *sativa*) from Siberia [26] and the sample from the seeds of the Romanian accession of *P. sativa* L. ssp. *sylvestris* [Mill.] Rouy and Camus (Syn: *P. sativa* ssp. *sativa*) [28], whereas octyl butanoate, the second most abundant metabolite in **PSS**, is the principal example in several cases [21,22,24,25,29,30]. On the other hand, hexyl butanoate, occurring in **PSS** in moderate amounts (7.9%), has been detected as a main product in the EOs from flowers and fruits of *P. hirsuta* from Serbia [17,22], in the sample obtained from aerial parts of *P. sativa* L. ssp. *sativa* from Serbia [18], and in the EO of the Serbian accession of *P. sativa* ssp. *urens* [22]. From a careful reading and interpretation of the data reported in Table 2, the major esters, octyl butanoate, octyl acetate, and hexyl butanoate, or the flavouring agent *γ*-palmitolactone itself, are abundant in fruits, leaves, and flowers, and therefore, in general, in the aerial parts. Small quantities (%) of these compounds were detected in the roots [22]. On the other hand, the phenylpropanoid myristic, present in our EO only for 2.9% of the total composition, was identified in abundance in the EOs obtained from the roots of the different *Pastinaca* specimens analysed [20,22,23,27], or even in the aerial parts of plants at the end of the vegetative cycle [17].

### 2.2. Antimicrobial Properties of **PSS**

**Figure 1:**
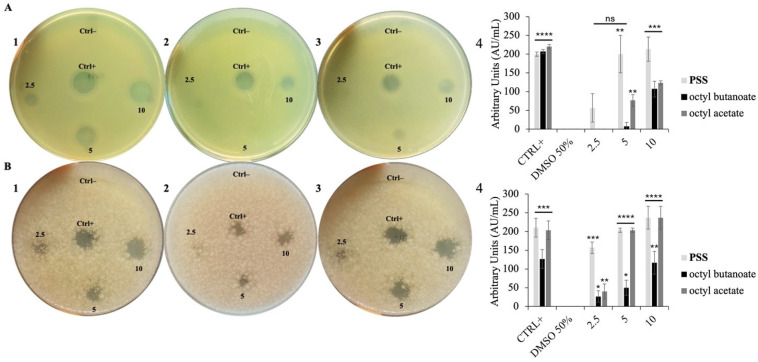
Kirby–Bauer Antimicrobial Assay. Panel (**A**): activity against *E. coli*; Panel (**B**): activity against *B. cereus*. Images 1, 2, and 3 in each panel correspond to inhibition zone plates: 1 = halo with **PSS**, 2 = halo with octyl butanoate, 3 = halo with octyl acetate. Image 4 represents the corresponding quantitative data in arbitrary units/mL. Treatments were tested with 2.5, 5, and 10 µL. Ampicillin and 50% DMSO served as positive and negative controls, respectively. Statistical analysis was performed using a two-tailed paired *t*-test vs. DMSO 50%, data = mean ± SD (n = 3); ns = not significant; *p* < 0.05 (*), <0.01 (**), <0.001 (***), <0.0001 (****).

To evaluate the antimicrobial activity of **PSS**, the Kirby–Bauer disk diffusion assay was initially employed as a preliminary screening method. This approach allowed us to assess the potential antimicrobial properties of the EO by measuring the presence and diameter of inhibition zones formed against *E. coli* and *B. cereus* (Figure 1).

As observed, **PSS** exhibited clear antimicrobial activity, with a notably stronger effect against the Gram-positive strain *B. cereus*, even at the lowest tested volume of 2.5 µL. Interestingly, among the individual esters, octyl acetate appeared to exert the most pronounced antimicrobial effect, consistent with reports that this compound often dominates the biological activity in related EOs [31]. This greater sensitivity of *B. cereus* compared to *E. coli* is consistent with previous studies demonstrating that Gram-positive bacteria are generally more susceptible to EO activity. This difference is often attributed to the structural characteristics of bacterial cell walls. Gram-negative bacteria possess an outer membrane that limits the diffusion of hydrophobic compounds, such as EO constituents [32].

Based on preliminary experiments indicating a greater sensitivity of Gram-positive bacteria to **PSS,** the MIC determination beyond the initially selected model strains was extended. In addition to *E. coli* and *B. cereus*, two more Gram-positive strains (*B. subtilis* and *M. smegmatis*) and two Gram-negative species (*P. aeruginosa* and *S. typhimurium*) were included, in order to obtain a more comprehensive profile of the antimicrobial activity of **PSS** and its individual components across diverse bacterial types.

As shown in Table 3, the lowest MIC values were observed against Gram-positive bacteria, ranging from 23,000 to 15,000 µg/mL for *B. subtilis*, *B. cereus*, and *M. smegmatis*, respectively. In contrast, the MIC values recorded for Gram-negative bacteria such as *E. coli* and *P. aeruginosa* were generally higher, averaging around 25,000 µg/mL, with *S. typhimurium* presenting mics greater than 25,000 µg/mL.

MICs were also determined for the individual components (octyl acetate and octyl butanoate).

These findings support the commonly observed trend that EOs, particularly those rich in esters and monoterpenes, tend to be more effective against Gram-positive bacteria. This enhanced susceptibility is likely due to the simpler cell wall structure of Gram-positive organisms, which lack the outer membrane barrier characteristic of Gram-negative species, allowing lipophilic EO components to diffuse more easily and exert their antimicrobial action [33]. These results align with the existing literature reporting the antimicrobial potential of *P. sativa* essential oils. For instance, Ušjak et al. [22] found that this EO displayed significant inhibitory effects, particularly against Gram-positive strains, suggesting the presence of active metabolites such as octyl butanoate and octyl acetate as key contributors. Similarly, Semerdjieva et al. [21] investigated the antimicrobial properties of ester-rich EOs from various Apiaceae species and found comparable MIC ranges, highlighting that esters like octyl acetate and octyl butanoate—major components in *P. sativa* EO—were likely responsible for the antibacterial effects observed.

Taken together, these findings validate the antimicrobial efficacy of **PSS** and underline its potential application as a natural antimicrobial agent, particularly in formulations aimed at controlling Gram-positive pathogens, including foodborne or opportunistic strains.

### 2.3. **PSS** Target Determination in Bacteria

Given that several EOs are widely reported to exert their antibacterial effects primarily through disruption of bacterial membranes, the potential mechanism of action of the **PSS,** and of individual components, has been investigated. Numerous studies suggest that EO constituents rich in esters and monoterpenes compounds can compromise the structural integrity of bacterial outer membranes (OM), leading to increased permeability and eventual cell lysis [34].

**Figure 2:**
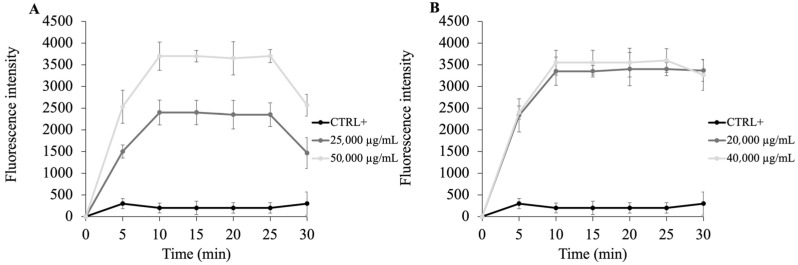
Outer Membrane Damage Assessed by NPN Assay. NPN fluorescence was used to evaluate outer membrane permeability in *E. coli* (Panel (**A**)) and *B. cereus* (Panel (**B**)) after treatment with MIC and 2 × MIC concentrations of **PSS**. Data represent the mean of three independent experiments.

To explore this mechanism, the fluorescent probe N-phenyl-1-naphthylamine (NPN), a hydrophobic dye with minimal fluorescence in aqueous environments, was initially used. When the OM remains intact, NPN is excluded and cannot interact with the lipid tails of the phospholipid bilayer. However, upon membrane disruption, NPN intercalates into hydrophobic regions, resulting in a sharp increase in fluorescence. Therefore, the fluorescence intensity of NPN can be used as an indirect measure of membrane permeabilization induced by antimicrobial agents [35]. As illustrated in Figure 2, panel A, **PSS,** at its MIC value, causes a modest increase in fluorescence emission in *E. coli* cells, with a slightly higher intensity observed at twice the MIC concentration (50,000 µg/mL). In contrast, panel B reveals that *B. cereus* cells exhibit a more pronounced fluorescence response, both at MIC and 2 × MIC, indicating a greater degree of membrane disruption compared to *E. coli*. Notably, the fluorescence values at MIC and 2 × MIC are relatively similar in *B. cereus*, yet higher than those observed in *E. coli* (respectively 20,000 and 40,000 µg/mL). This suggests that in *B. cereus*, even the MIC concentration is sufficient to rapidly compromise membrane integrity, allowing NPN uptake, whereas in *E. coli* a significant increase in fluorescence is only evident at 2 × MIC, likely due to the additional protective barrier of the outer membrane already at 5 min. These findings suggest that **PSS** exerts a membrane-targeting antimicrobial effect. This differential sensitivity may be attributed to the simpler membrane architecture of Gram-positive species, which lack the protective outer membrane present in Gram-negative bacteria [32].

Comparable observations have been reported in studies evaluating the mechanism of action of other *Apiaceae* EOs. For example, Kurkcuoglu et al. [29] demonstrated that samples rich in esters could destabilize bacterial membranes, thereby enhancing permeability and inducing cell death [29]. Similarly, Semerdjieva et al. [21] found that ester-dominant EOs exhibited strong membrane-perturbing effects, particularly against Gram-positive strains.

In conclusion, NPN uptake results support the hypothesis that the antimicrobial activity of **PSS** is largely mediated by its ability to compromise membrane integrity, particularly in Gram-positive bacteria, aligning with previously published mechanistic studies on essential oil components.

To better elucidate the biological mechanism of **PSS**, the biological activity of its main constituents—octyl acetate, octyl butanoate, and hexyl butanoate—was investigated individually. These compounds showed moderate antimicrobial effects when tested individually; however, their combination reproduced the broader spectrum and higher efficacy observed with the whole EO, supporting the probable hypothesis of synergistic or additive interactions between the components. This is in line with previously reported structure–activity relationship (SAR) studies on aliphatic esters, showing that antimicrobial activity tends to increase with alkyl chain length due to increased lipophilicity and membrane penetration [36,37]. In particular, medium-chain esters (C_6_–C_10_) are known to effectively integrate and disrupt bacterial membranes, contributing to their antimicrobial potency [38].

**Figure 3:**
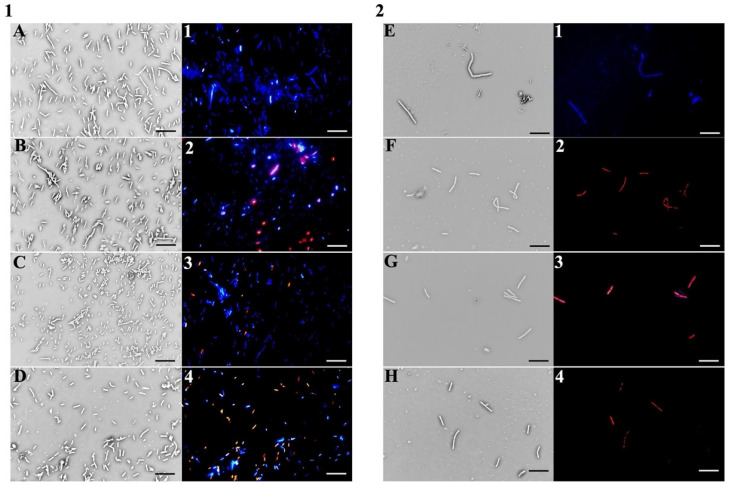
Fluorescence microscopy of *E. coli* (panel (**1**)) and *B. cereus* (panel (**2**)) cells after 30 min of treatment with **PSS** (**B**,**F**,**1-2**,**2-2**), octyl butanoate (**C**,**G**,**1-3**,**2-3**), and octyl acetate (**D**,**H**,**1-4**,**2-4**), stained with DAPI (blue) and propidium iodide (red). Untreated control cells are shown in (**A**,**E**,**1-1**,**2-1**). Scale bars: 5 µm.

To further confirm that the bacterial membrane is the cellular target of **PSS**, fluorescence staining using the DAPI/propidium iodide (PI) double labelling method was performed after 30 min of incubation at MIC values with both **PSS** and its individual components. DAPI (4′,6-diamidino-2-phenylindole) is a cell-permeable fluorescent stain that emits blue fluorescence when bound to DNA. In contrast, propidium iodide is membrane-impermeable and penetrates only cells with damaged membranes, where it intercalates into DNA and fluoresces red. Thus, red fluorescence clearly indicates membrane disruption and loss of cell viability. As shown in Figure 3, panel 1 (*E. coli* cells), treatment with **PSS** (B, 2), octyl butanoate (C, 3), and octyl acetate (D, 4) led to increased red fluorescence, especially in cells treated with pure **PSS** and octyl acetate. Panel 2, showing *B. cereus* cells under the same treatment conditions with **PSS** (F, 2), octyl butanoate (G, 3), and octyl acetate (H, 4), exhibited marked red fluorescence in all samples, indicating compromised membranes.

Fluorescence microscopy using DAPI and propidium iodide confirmed that the antimicrobial effect of **PSS** and its major ester components is associated with disruption of bacterial membrane integrity. The pronounced red fluorescence observed, particularly in *B. cereus* samples, indicates extensive membrane damage and a higher degree of permeability compared to *E. coli*. These findings, consistent with the NPN uptake results, suggest that Gram-positive bacteria are more susceptible to the pure EO and its components. Overall, the new data confirm that membrane disruption is a central mechanism of action, consistent with the chemical profile of **PSS** and published SAR data.

### 2.4. Antibiofilm Activity of **PSS**

Given the antibiofilm activity of various EOs at sub-inhibitory concentrations [16], it was hypothesized that the **PSS** might exhibit similar effects. To test this hypothesis, experiments were conducted, initially using *M. smegmatis*, a non-pathogenic model organism frequently employed in mycobacterial research due to its genetic and physiological similarities to *Mycobacterium tuberculosis*.

Considering that the minimum inhibitory concentration (MIC) of **PSS** against *M. smegmatis* was previously determined to be 15,000 µg/mL, concentrations were investigated ranging from 0 to 2500 µg/mL to assess the EO’s capacity to inhibit biofilm formation.

**Figure 4:**
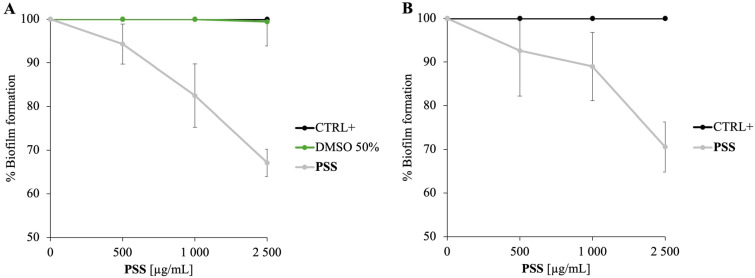
Inhibition of biofilm formation by **PSS** on *M. smegmatis* (Panel (**A**)) and *P. aeruginosa* (Panel (**B**)). Biofilm biomass was quantified after treatment with concentrations included between 0 and 2500 µg/mL of **PSS**. The results show a dose-dependent inhibition. Data represent the mean ± SD of three independent experiments.

As illustrated in Figure 4A, treatment with **PSS** at 2500 µg/mL dose resulted in approximately 30% inhibition of biofilm formation. This finding is noteworthy, as it suggests that **PSS** possesses the ability to interfere with the initial stages of biofilm development, a critical process in bacterial colonization and infection persistence.

The antibiofilm activity observed at concentrations that do not cause bacterial death aligns with previous studies demonstrating that some EOs can inhibit biofilm formation without exerting bactericidal effects, thereby reducing selective pressure for resistance development [39]. This antibiofilm effect may result from disruption of cell membrane integrity or interference with quorum sensing pathways, mechanisms reported for other EOs rich in aliphatic esters [40]. So, although **PSS** has antimicrobial potential, its ability to inhibit biofilm formation at low concentrations is not attributable to bacterial killing but rather to interference with initial adhesion and biofilm development processes. Therefore, the biofilm inhibition observed under these conditions results from a non-lethal, anti-adhesive or quorum sensing-disrupting effect rather than direct bactericidal activity. This dual behaviour of EOs (bactericidal at higher concentrations and biofilm-inhibitory at sub-lethal levels) has also been reported in the literature [32,41] and is a well-recognized feature of many EOs.

**Figure 5:**
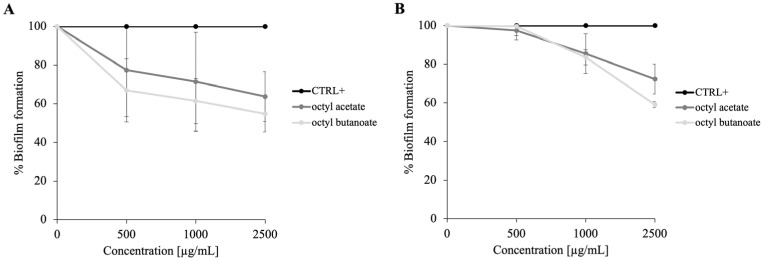
Inhibition of biofilm formation by octyl acetate and octyl butanoate on *M. smegmatis* (Panel (**A**)) and *P. aeruginosa* (Panel (**B**)). Biofilm biomass was quantified after treatment with concentrations included of between 0 and 2500 µg/mL of these compounds. The results show a dose-dependent inhibition. Data represent the mean ± SD of three independent experiments.

To clarify this aspect, the antibiofilm activity of **PSS** was also tested against *P. aeruginosa*, a model organism extensively studied for quorum sensing-regulated biofilm formation (Figure 4B). The data confirmed that **PSS** exhibits promising antibiofilm effects against *P. aeruginosa*, supporting the hypothesis that interference with quorum sensing contributes to this activity. While quorum sensing mechanisms are well established in *P. aeruginosa*, their role in *M. smegmatis* remains less clear and warrants further investigation. Given that octyl acetate and octyl butanoate are the main constituents of **PSS**, their roles in modulating biofilm formation were further investigated. Therefore, to define the contribution of individual components, the antibiofilm activities of octyl acetate and octyl butanoate were evaluated on both *M. smegmatis* and *P. aeruginosa* (Figure 5).

Panel A shows the effect on *M. smegmatis* biofilm, and panel B shows the corresponding effects on *P. aeruginosa*. In both cases, octyl butanoate demonstrated a stronger inhibitory effect, reducing biofilm formation by approximately 40%. These results suggest that, among the volatile constituents, octyl butanoate may play a major role in the antibiofilm properties of the whole **PSS**. This selective activity could be due to specific interactions with bacterial cell envelopes or interference with early biofilm developmental signals. Overall, these complementary findings reinforce the potential of plant-derived EOs as promising alternative strategies to combat biofilm-associated infections caused by diverse bacterial species.

### 2.5. Cytotoxic Activity of **PSS**

**Figure 6:**
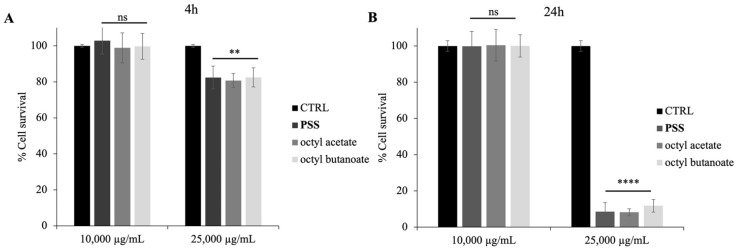
Cytotoxicity of **PSS**, octyl acetate and octyl butanoate on Caco-2 cells assessed by MTT assay. Panel (**A**) shows cell viability after 4 h of treatment; Panel (**B**) shows results after 24 h. Cells were treated with 10,000 and 25,000 µg/mL. Data are presented as mean ± SD of three independent experiments. Statistical analysis was performed by two-tailed paired *t*-test relative to untreated cells: ns = not significant; *p* < 0.01 (**), *p* < 0.0001 (****).

To verify whether the **PSS** could be toxic to eukaryotic cells, human intestine cells (Caco-2) were used to perform an assay using the MTT reagent, as reported in the methods. The MTT assay revealed a dose- and time-dependent effect of **PSS**, octyl acetate and octyl butanoate on cell viability. At the 4-h time point, cell survival remained high (approximately 100%) in both the control group and the group treated with 10,000 µg/mL (Figure 6A). However, a significant reduction in cell survival to approximately 80% was observed at the 25,000 µg/mL **PSS** concentration. This cytotoxic effect of the higher **PSS** and its individual components’ concentration became more pronounced after 24 h of exposure, where cell survival was drastically reduced to below 20% at 25,000 µg/mL (Figure 6B). In contrast, cell survival at 10,000 µg/mL **PSS** remained near 100% after 24 h, similar to that of the control group. These results indicate that **PSS** and its individual components exhibit no detectable cytotoxicity at 10,000 µg/mL, while higher concentrations (25,000 µg/mL) significantly reduce cell viability, particularly with prolonged exposure, for 24 h. This is in line with previous literature on other *Pastinaca* species, where concentrations up to 10,000 µg/mL were also found to be non-toxic on the same Caco-2 cell line used in our experiments [42].

### 2.6. Effect of **PSS** on Cell Proliferation

**Figure 7:**
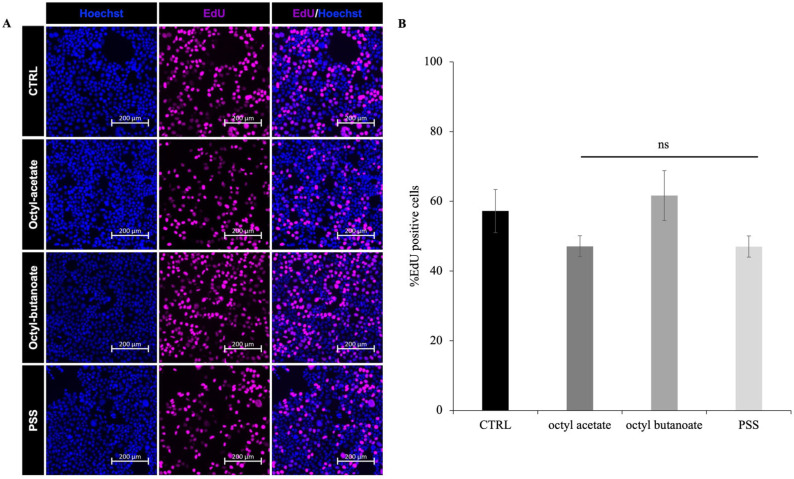
Effect of **PSS,** octyl acetate and octyl butanoate on cell proliferation in Caco-2 cells assessed by EdU incorporation. (**A**) Caco-2 cells were pulsed with 10 mM EdU 3 h before fixation and stained 24 h after **PSS** treatment. Detection by immunofluorescence analysis of proliferating EdU positive cells (purple) and total nuclei (Hoechst in blue). (**B**) Percentage of EdU+ nuclei/total number of nuclei in Caco-2 cells treated with 10,000 µg/mL of **PSS** octyl acetate and octyl butanoate for 24 h Data are presented as mean ± SD of three independent experiments. Statistical analysis was performed by two-tailed paired *t*-test relative to untreated cells: ns = not significant.

Based on the observed reduction in cell viability following **PSS,** octyl acetate and octyl butanoate, treatment in the MTT assay, the effect was investigated at the lowest concentration (10 μg/mL) on cell proliferation using the Click-iT EdU Cell Proliferation Kit. This proliferation detection assay is based on the incorporation and immunofluorescence measurement of the thymidine analog 5-ethynyl-2′-deoxyuridine (EdU) into newly synthesized DNA during *S* phase. Caco-2 cells were treated with 10,000 µg/mL **PSS**, octyl acetate and octyl butanoate, for 24 h, and EdU was added to the cell culture medium three h before fixation. Immunofluorescent analysis of Hoechst (nuclear marker) and EdU (proliferation marker) staining revealed a similar percentage of EdU-positive cells in **PSS**, octyl acetate and octyl butanoate-treated cells compared to the control group (CTRL) (Figure 7A). Quantitatively, the percentage of EdU-positive cells was approximately 50% in both the control and treated groups (Figure 7B). These results indicate that treatment with **PSS** and its individual components, under the conditions of this experiment, does not significantly influence the cell proliferation rate as measured by EdU incorporation. An EO that does not affect cell proliferation is generally defined as a non-cytotoxic or non-proliferative agent, as reported for **PSS** up to a concentration of 10,000 µg/mL.

### 2.7. Antioxidant Activity of **PSS** on Eukaryotic Cells

**Figure 8:**
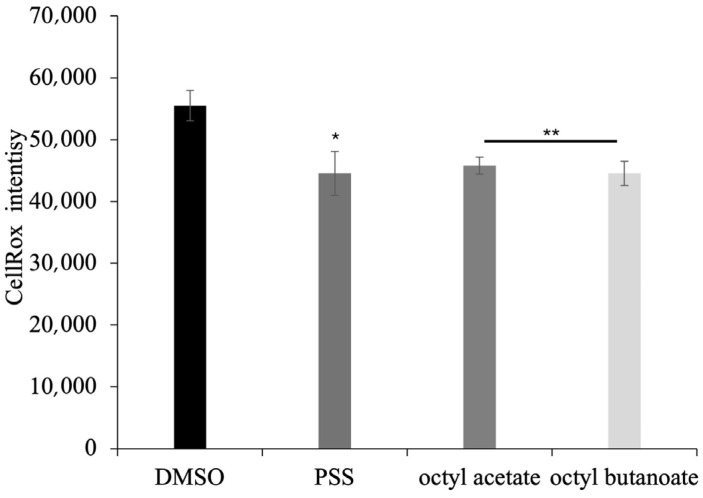
Effect of **PSS**, octyl acetate and octyl butanoate on H_2_O_2_-induced ROS generation in Caco-2 cells. Oxidative stress determination using CellROX fluorescent dye. Caco-2 cells were treated with 800 μM H_2_O_2_ 3 h before ROS detection. 5 μM of CellROX was added to Caco-2 cells for 30 min and cells were analysed 24 h after pre-treatment. Data are presented as mean ± SD of three independent experiments. Statistical analysis was performed by two-tailed paired *t*-test relative to negative control DMSO: *p* < 0.05 (*); *p* < 0.01 (**).

ROS-sensitive fluorescent dye was used to investigate whether the non-toxic concentration of **PSS,** octyl acetate and octyl butanoate (10,000 µg/mL) prevent H_2_O_2_-induced ROS generation. Caco-2 cells that had been exposed to H_2_O_2_ showed a significant increase in the accumulation of intracellular ROS, whereas this induction was significantly inhibited by the **PSS** pre-treatment (Figure 8). Accordingly, recent studies revealed an increased antioxidant effect of **PSS** in Caco-2 cells in a dose-dependent manner, as observed in this study [43].

## 3. Materials and Methods

### 3.1. Plant Material

The aerial parts from *Pastinaca sativa* subsp. *sativa* were taken from near San Candido, Bolzano, South Tyrol (Italy) (46°43′56″ N; 12°17′38″ E, 1260 m s/l.) (Figure S1), in August 2024. One of the samples, identified by Prof. Vincenzo Ilardi, has been stored in the Herbarium Mediterraneum Panormitanum (PAL) (Voucher N.109996), of the Botanical Garden of the University of Palermo, Italy.

### 3.2. Isolation of EO

Fresh full aerial parts (flowers, stems, and leaves), and a small part of the roots (≈250 g) were subjected to hydro-distillation for 3 h, according to the standard procedure described in the European Pharmacopoeia [44]. Samples yielded 0.15% of EO.

### 3.3. GC-MS Analysis

Chemical analysis of EO was performed on a Shimadzu QP 2010 plus equipped with an AOC-20i autoinjector (Shimadzu, Kyoto, Japan) gas chromatograph with a capillary column (DB-5 MS) 30 m × 0.25 mm i.d., film thickness 0.25 μm and a data processor. The oven temperature programme was the following: 5 min at 40 °C, subsequently 2 °C/min up to 260 °C, then isothermal for 20 min. Injector and detector temperatures were 250 and 280 °C, respectively. He was used as the carrier gas, at a flow rate of 1 mL/min. Split ratio, 1:50; acquisition mass range, *m*/*z* 40–400. All mass spectra were acquired in electron-impact (EI) mode with an ionization voltage of 70 eV. The GC conditions were the same as those reported for GC–MS analyses. The pressure was 35 kPa at pressure constant. The carrier gas was it, and u was 32.12 cm/s. The injection volume was 1.0 μL. The split ratio was 1:50, ionization voltage was 70 kV, and acquisition mass range was 40–400 *m*/*z*. The area percentage calculation (%) was calculated by dividing the area of each individual peak by the total area of all peaks in the chromatogram and then multiplying by 100. Area percentages are used to determine the relative abundance of each component in the essential oil. The settings were as follows: ionization voltage, 70 eV; electron multiplier energy, 2000 V; transfer line temperature, 295 °C; solvent delay 3 min. Linear retention indices (LRI) were determined by using retention times of *n*-alkanes (C_8_–C_40_) and the peaks were identified by comparison with mass spectra, by comparison of their relative retention indices with WILEY275, NIST 17, ADAMS, and FFNSC2 libraries, and by comparing mass, retention index and time of co-injected octyl acetate and octyl butanoate.

### 3.4. Pure Compounds

Pure compounds, octyl acetate and octyl butanoate were purchased from Sigma-Aldrich (Sigma-Aldrich Chemie GmbH, Eschenstr. 5, 82024 Taufkirchen, Germany).

### 3.5. Microorganisms

The antimicrobial activity of **PSS**, octyl acetate and octyl butanoate was evaluated against different strains: *Escherichia coli* DH5α, *Pseudomonas aeruginosa* PAO1 ATCC15692 and *Salmonella typhimurium* ATCC14028 (Gram-negative) and *Bacillus cereus* ATCC10987, *Bacillus subtilis* AZ54 and *Mycobacterium smegmatis* MC^2^155 (Gram-positive).

### 3.6. Sample Preparation

To ensure full solubilization of the compound, stock solutions of the test samples were prepared in 50% (*v*/*v*) DMSO. Prior to use in any biological assay, these stocks were appropriately diluted in the corresponding test medium. In all experimental conditions, the final DMSO concentration never exceeded ≤2% (*v*/*v*), a concentration widely regarded as non-inhibitory to bacterial growth. This approach ensured that any observed antimicrobial effects could be attributed solely to the tested compounds rather than to the solvent.

### 3.7. Kirby–Bauer Disk Diffusion Assay

The potential antimicrobial activity of **PSS**, octyl acetate and octyl butanoate was initially assessed using a modified version of the Kirby–Bauer disk diffusion method [45]. Volumes of 2.5, 5, and 10 μL of samples were applied as droplets onto Luria–Bertani (LB) agar plates overlaid with 10 mL of soft agar (0.7%) inoculated with 10 μL of *E. coli* or *B. cereus* cultures previously incubated for 24 h at 37 °C. A 50% DMSO solution, used to dissolve the EO, served as a negative control, whereas ampicillin was employed as a positive control. Plates were incubated overnight at 37 °C, and antimicrobial activity was assessed by measuring the diameter of inhibition zones in millimeters [16].

The antimicrobial units (AU/mL) were calculated using the following formula:

$$\frac{Diameter\;of\;the\;zone\;of\;clearance\left(mm\right)\times 1000}{Volume\;applied\;to\;the\;well(\mu L)}$$

### 3.8. Determination of Minimum Inhibitory Concentrations (MIC)

The antimicrobial activity of **PSS**, octyl acetate and octyl butanoate was assessed by determining the minimum inhibitory concentrations (MIC) against selected bacterial strains using the broth microdilution method, following the guidelines of the Clinical and Laboratory Standards Institute (CLSI) [46].

Briefly, bacterial cultures were grown overnight, adjusted to a final concentration of 5 × 10^5^ CFU/mL, and added to 96-well microplates containing 195 µL of Mueller–Hinton broth (CAM-HB; Difco). **PSS**, octyl acetate and octyl butanoate were diluted in DMSO 50% and tested in concentrations ranging from 1000 to 30,000 µg/mL. After inoculation, plates were incubated at 37 °C for one night.

Bacterial growth was evaluated by measuring the optical density at 600 nm using a microplate reader. MIC was defined as the lowest concentration of the essential oil that completely inhibited visible growth.

### 3.9. N-Phenyl Naphthylamine (NPN) Assay

The outer membrane (OM) permeabilizing activity of **PSS** was assessed using the 1-N-phenylnaphthylamine (NPN) uptake assay, with slight modifications based on the protocol described by Jia et al. [47]. Briefly, *E. coli* and *B. cereus* cells were harvested after overnight incubation under their optimal growth conditions, washed with PBS, and resuspended in 5 mM HEPES buffer (pH 7.2) to an optical density at 600 nm of 0.5 ± 0.02.

For the assay, 50 µL of **PSS** at concentrations corresponding to the MIC and twice the MIC were added to 100 µL of bacterial suspension in black 96-well fluorescence microplates. Subsequently, 50 µL of NPN was added to reach a final concentration of 40 µM. Control wells contained only HEPES buffer, bacterial cells, and NPN without **PSS**. Fluorescence measurements were taken using a fluorometer with excitation and emission wavelengths set at 350 nm and 420 nm, respectively.

The fluorescence intensity was recorded as an indicator of outer membrane permeabilization.

### 3.10. Fluorescence-Based Viability Assay Using DAPI and PI

To evaluate bacterial membrane integrity through fluorescence microscopy, two fluorescent dyes were used: DAPI (4′,6-diamidino-2-phenylindole dihydrochloride; Sigma-Aldrich, Milan, Italy) and propidium iodide (PI; Sigma-Aldrich, Milan, Italy). In brief, 100 µL of bacterial suspensions (*E. coli* and *B. cereus* reference strains) were incubated at 37 °C for 30 min in the dark with shaking, either untreated or treated with **PSS** or its main constituents, octyl acetate and octyl butanoate, at their respective MIC values.

After incubation, 10 µL of each sample was combined with a staining solution containing DAPI (1 µg/mL) and PI (20 µg/mL). Fluorescence imaging was performed using an Olympus BX51 microscope (Olympus, Tokyo, Japan) equipped with a DAPI-specific filter set (excitation/emission: 358/461 nm). The exposure time for dual DAPI/PI staining was standardized to 1000 ms. Images were acquired using an Olympus DP70 digital camera, following the method outlined by Di Napoli et al. [48].

### 3.11. Inhibition of Biofilm Development Assays

The antibiofilm activity of **PSS** and its individual components was assessed against *M. smegmatis* and *P. aeruginosa* biofilms using the crystal violet colorimetric assay. Untreated microbial cells served as positive controls, while cultures treated with 50% DMSO were used as negative controls. A 24-well plate was prepared and incubated at 37 °C for 72 h in the presence of **PSS** and its individual components at concentrations ranging from 0 to 2500 µg/mL to evaluate its ability to inhibit biofilm formation. Following incubation, biofilm biomass was quantified by staining with crystal violet, and absorbance was measured at 570 nm using a Multiskan microplate reader (Thermo Electron Corporation, Waltham, MA, USA) [49]. To account for variations in planktonic growth, results were normalized by calculating the ratio between the optical density at 570 nm (biofilm biomass) and the OD at 600 nm (planktonic cell density). The percentage of biofilm inhibition was determined by comparing normalized OD values of treated samples to those of untreated controls.

### 3.12. Eukaryotic Cell Culture

Caco-2 (colorectal adenocarcinoma) cells were maintained in Dulbecco’s Modified Eagle Medium (DMEM) (61965-026, GIBCO, Grand Island, NY, USA) supplemented with 10% FBS (Euroclone, Pero, Italy, ECS500L) 1%, Penicillin-Streptomycin (ECB3001D, Euroclone), 4 mM L-Glutamine (ECB3000D, Euroclone). Cells were grown at 37 °C in a humidified atmosphere of 5% (*v*/*v*) CO_2_.

### 3.13. MTT Assay

Caco-2 cells were seeded into 96-well plates. At 24 h after seeding, cells were treated with **PSS** and its individual components for 4 and 24 h, at the indicated concentrations, followed by standard MTT assay (abcam). Cells were incubated for 3 h at 37 °C in the dark with media containing 500 µg/mL of MTT solution. Afterward, the medium was replaced with isopropanol and incubated for another 30 min at 37 °C. Then, absorbance at 570 and 620 nm was determined using EnVision plate reader (Menlo Park, CA, USA).

### 3.14. Oxidative Stress Measurement

Caco-2 cells were seeded into 96-well plates. 24 h after seeding, cells were treated with 10,000 µg/mL of **PSS**, octyl acetate and octyl butanoate for 24 h. Cells were exposed to H_2_O_2_ (800 μM) for 3 h, followed by CellROX Green Reagent fluorogenic probe detection for measuring oxidative stress in live cells (Invitrogen, Carlsbad, CA, USA, C10444). Cells were incubated for 30 min at 37 °C in the dark with media containing 5 μM of CellROX solution. Afterward, medium was replaced with fresh medium. Then absorbance at 488 nm was determined using CellDiscoverer CD7 Zeiss (San Francisco, CA, USA).

### 3.15. EdU Incorporation Assay

To assess proliferation, Caco-2 cells were pulsed with 1 mM of EdU (ThermoFisher, Waltham, MA, USA, C10640) in growth medium for 3 h at 37 °C before fixation. Cells were rinsed twice with 1X PBS and fixed with warm 4% paraformaldehyde for 10 min at room temperature. EdU was revealed with Click-iT reaction according to manufacturer instruction.

## 4. Conclusions

The present study focused on the determination of the chemical composition and biological ability of the *Pastinaca sativa* subsp. *sativa* essential oil to exert a wide range of biological activities. For the first time, the chemical composition of the essential oil from a spontaneous Northern Italian population of *Pastinaca sativa* subsp. *sativa* was investigated and compared with the results obtained from species investigated in Serbia and Bulgaria. The analyses showed that, despite the geographical distance, this species presents as typical markers medium and long chain esters, such as octyl acetate and octyl butanoate. The essential oil was also investigated biologically, revealing to be a highly promising natural product not only as an antimicrobial, but also for its biofilm and antioxidant properties. Its antimicrobial efficacy was particularly pronounced against Gram-positive bacteria, likely due to its interaction with and disruption of the bacterial cell membrane, a known vulnerability in Gram-positive strains due to their simpler envelope structure. In addition, the two most relevant constituents—octyl acetate and octyl butanoate—demonstrated comparable antimicrobial, antibiofilm, and antioxidant properties, highlighting their key role in the overall bioactivity of the complete essential oil. Their ability to reproduce the biological effects of the total oil strongly suggests that they are the major contributors to its efficacy, particularly regarding antimicrobial action. Overall, the multifaceted bioactivity of *Pastinaca sativa* subsp. *sativa* essential oil as well as of its main active components octyl acetate and octyl butanoate, positions it as a strong candidate for further investigation as a natural agent for pharmaceutical, food preservation, or cosmetic purposes.

## Figures and Tables

**Table 1:** Chemical composition of *P. sativa* subsp. *sativa* EO (**PSS**).

No.	Compound ^a^	LRI ^b^	LRI ^c^	Relative Area (%) ^d^
1	1-Hexanol	865	865	0.1
2	*α*-Pinene	929	931	0.1
3	*β*-Pinene	970	969	0.2
4	Butyl butanoate	996	997	0.6
5	Octanal	1002	1004	1.0
6	Limonene	1024	1025	0.3
7	*trans*-Ocimene	1035	1037	1.3
8	*cis*-Ocimene	1047	1051	0.9
9	*γ*-Terpinene	1084	1089	0.1
10	*cis*-5-Octen-1-ol	1061	1057	0.2
11	1-Octanol	1072	1068	5.5
12	Terpinolene	1084	1089	0.4
13	Hexyl butanoate	1194	1197	7.9
14	Decanal	1204	1204	0.3
15	Octyl acetate	1217	1221	38.7
16	*α*-Terpinyl acetate	1344	1337	2.3
17	Octyl butanoate	1393	1396	26.7
18	Decyl acetate	1410	1413	0.7
19	Caryophyllene	1427	1429	0.3
20	*β*-Phenylethyl butyrate	1437	1441	1.0
21	*β*-Farnesene	1456	1458	1.1
22	*α*-Amorphene	1475	1479	0.2
23	Methylisoeugenol	1495	1492	0.9
24	*α*-Farnese	1506	1509	0.1
25	Myristicin	1516	1520	2.9
26	*trans*-Nerolidol	1556	1560	0.2
27	Octyl hexanoate	1581	1578	0.7
28	Phenethyl hexanoate	1639	1628	0.1
29	*γ*-Palmitolactone	2178	2185	0.6
	**Monoterpene Hydrocarbons**			**3.3**
	**Oxygenated Monoterpenes**			**2.3**
	**Sesquiterpene Hydrocarbons**			**1.9**
	**Alcohols**			**5.8**
	**Esters**			**77.3**
	**Other Compounds**			**4.8**
	**Total Composition**			**95.4**

^a^ Compounds are classified in order of linear retention time on the non-polar column (DB-5 MS); ^b^ Experimental LRIs on a DB-5 MS non-polar column; ^c^ LRIs on DB-5 MS column reported in the literature; ^d^ Area is the peak volume percentage of compound in the EO sample.

**Table 2:** Main constituents (>3%) of the EOs, obtained by HD, of *Pastinaca* taxa, reported in the literature.

Taxa	Origin and Sample Parts	Compounds	References
*P. hirsuta* Pančić.	Serbia, roots at the flowering stage	apiole (33.4%), (*Z*)-falcarinol (22.7%), myristicin (17.0%), *β*-bisabolene (4.9%), *γ*-palmitolactone (3.6%)	[17]
	Serbia, roots at the fruiting stage	apiole (45.3%), myristicin (30.1%), (*Z*)-falcarinol (8.1%), terpinolene (3.1%)	[17]
	Serbia, stems at the flowering stage,	*γ*-palmitolactone (51.9%), propyl linoleate (23.3%), (*E*)-*β*-ocimene (8.6%), methyl linoleate (3.0%)	[17]
	Serbia, stems at the fruiting stage	*γ*-palmitolactone (45.7%), propyl linoleate (16.7%), (*E*)-*β*-ocimene (8.1%), methyl linoleate (4.8%), (*E*)-nerolidol (3.2%)	[17]
	Serbia, flowers	hexyl butanoate (31.1%), hexyl hexanoate (15.9%), *γ*-palmitolactone (13.6%) (*E*)-*β*-ocimene (8.7%), propyl linoleate (5.3%), terpinolene (3.4%)	[17]
	Serbia, fruits	hexyl butanoate (80.4%), hexyl hexanoate (12.1%)	[17]
	Serbia, aerial parts	(*Z*)-*β*-ocimene (10.8%), hexyl butanoate (10.4%), (*E*)-*β*-farnesene (6.1%), lavandulyl acetate (5.2%), (+)-*γ*-terpinene (3.7%), germacrene D (3.7%), *ar*-curcumene (3.3%), *α*-zingiberene (3.3%)	[18,19]
	Serbia, roots	apiole (56.0%), myristicin (21.0%), *β*-bisabolene (7.2%)	[20]
	Serbia, aerial parts	hexyl hexanoate (59.8%), hexyl butanoate (21.4%)	[20]
	Bulgaria, Loc 1, aerial parts	*n*-octyl butanoate (46.5%), *n*-hexyl butanoate (16.0%), *n*-tricosane (10.7%), guaiol (7.2%), *n*-octanal (3.2%)	[21]
	Bulgaria, Loc 2, aerial parts	neryl acetate (28.4%), (*Z*)-hexenyl benzoate (5.4%), germacrene D-4-ol (4.3%), nerol (4.3%), α-pinene (4.1%), *β*-himachalene (3.8%), geranyl butanoate (3.1%), neryl propanoate (3.1%), italicene (3.1%)	[21]
	Bulgaria, Loc 2, flowers	neryl acetate (24.9%), tetrahydro-lavandulol acetate (13.4%), neryl propanoate (5.7%), *α*-terpinyl acetate (5.6%), (*E*,*E*)-*α*-farnesene (5.6%), italicene (4.1%), (2*E*)-tridecenol (3.7%), guaiol (3.7%), *n*-hexyl butanoate (3.6%), lavandulyl isobutanoate (3.1%)	[21]
	Bulgaria, Loc 2, seeds	neryl acetate (15.9%), *n*-tricosane (15.2%), tetrahydro-lavandulol acetate (10.6%), neryl propanoate (8.8%), geranyl butanoate (4.5%), α-pinene (3.6%), *n*-octadecane (3.3%)	[21]
	Serbia, flowers	hexyl butanoate (61.9%), hexyl hexanoate (17.0%), *γ*-palmitolactone (6.0%)	[22]
	Serbia, fruits	hexyl butanoate (22.9–58.4%), hexyl hexanoate (29.1–59.8%), octyl acetate (1.5–3.3%)	[22]
	Serbia, leaves	*γ*-palmitolactone (47.5%), octadecadienoic acid (24.3%), *β*-pinene (3.9%), (*E*)-*β*-ocimene (3.9%)	[22]
	Serbia, stems	*γ*-palmitolactone (53.3–60.4%), octadecadienoic acid (25.5–34.0%), hexadecanoic acid (2.8–4.1%)	[22]
	Serbia, roots	apiole (25.8–30.9%), (*Z*)-falcarinol (12.2–25.9%), myristicin (11.6–20.3%), *γ*-palmitolactone (7.9–12.4%), octadecadienoic acid (5.0–6.7%), *β*-bisabolene (2.4–4.0%)	[22]
*P. sativa* L. ssp. *sativa*	Serbia, aerial parts	hexyl butanoate (55.4%)	[18]
	Germany, roots	terpinolene (40.3–69.0%), myristicin (17.2–40.1%), *β*-pinene (2.4–8.6%), limonene (1.7–3.2%), (*Z*)-*β*-ocimene (0.7–3.7%)	[23]
	Germany, fruits	octyl butanoate (46.2%), octyl acetate (32.8%), *n*-hexyl butanoate (6.4%)	[24]
	Germany, leaves	(*Z*)-*β*-ocimene (18.3%), (*E*)-*β*-farnesene (17.1%), *γ*-palmitolactone (16.2%), (*E*)-*β*-ocimene (12.6%)	[24]
	Germany, petiole	(*Z*)-*β*-ocimene (40.6%), *γ*-palmitolactone (18.3%), (*E*)-*β*-ocimene (17.1%), (*E*)-*β*-farnesene (7.2%), terpinolene (5.3%)	[24]
	Germany, stems	(*Z*)-*β*-ocimene (30.5%), terpinolene (22.6%), *γ*-palmitolactone (15.1%), (*E*)-*β*-ocimene (14.4%), (*E*)-*β*-farnesene (5.0%)	[24]
	Serbia, cult. flowers	octyl butanoate (31.4%), myristicin (21.5%), (*E*)-*β*-farnesene (10.3%), *γ*-palmitolactone (7.9%), (*Z*)-*β*-ocimene (3.7%)	[22]
	Serbia, cult. fruits	octyl butanoate (70.9–79.0%), octyl hexanoate (6.5–8.1%), *n*-octanol (1.4–9.1%), octyl acetate (0.6–5.1%), myristicin (0.5–3.8%)	[22]
	Serbia, cult. leaves	myristicin (41.4–42.8%), (*E*)-*β*-farnesene (22.3–22.4%), (*Z*)-*β*-ocimene (2.8–9.0%), *α*-(*E*)-bergamotene (6.6–7.7%), (*E*)-*β*-ocimene (1.5–3.8%), germacrene D (2.0–3.3%), *γ*-palmitolactone (1.2–3.2%)	[22]
	Serbia, cult. stems	myristicin (63.3–64.9%), *γ*-palmitolactone (11.8–18.4%), (*E*)-*β*-farnesene (7.0–14.4%)	[22]
	Serbia, cult. roots	myristicin (59.3–82.5%), terpinolene (1.2–28.7%), *β*-pinene (0.1–4.6%), *γ*-palmitolactone (0–4.5%), (*Z*)-falcarinol (0–3.6%)	[22]
*P. silvestris* Mill. (Syn: *P. sativa* ssp. *sativa*)	Siberia, seeds	*n*-octyl butanoate (32.0%), octyl acetate (27.0%), *Z*-asarone (14.1), *n*-hexyl butanoate (5.2%), *n*-octyl hexanoate (4.2%)	[25]
	Siberia, seeds	phytol (48.7%), (*E*)-*β*-farnesene (12.1%), *trans*-muurola-3,5-dien (10.8%)	[26]
	Siberia, leaves	(*E*)-*β*-farnesene (17.8%), (*E*)-caryophyllene (16.0%), *cis*-*β*-ocimene (10.7%)	[26]
	Siberia, flowers	octyl acetate (17.8%), *cis*-*β*-ocimene (16.0%), (*E*)-*β*-farnesene (11.3%)	[26]
*P. sativa* ssp. *sativa* var. *hortensis* (Syn: *P. sativa* ssp. *sativa*)	Cultivated, roots	terpinolene (36.0%), myristicin (24.7%), apiole (22.9%), dill-apiole (5.3%)	[27]
*P. sativa* L. ssp. *sylvestris* [Mill.] Rouy and Camus (Syn: *P. sativa* ssp. *sativa*)	Romania, seeds	octyl acetate (78.5%), octyl hexanoate (6.7%)	[28]
*P. sativa* L. subsp. *urens* (Req. ex Godron) Celak.	Turkey, aerial parts	octyl butanoate (79.5%), octyl hexanoate (5.3%), hexyl butanoate (3.3%)	[29]
	Turkey, fruits	octyl butanoate (90.4%)	[30]
	Turkey, aerial parts	*cis*-*β*-ocimene (38.2%), octadecanoic acid (14.1%), octyl butanoate (13.2%), butanoic acid (11.1%), *trans*-*β*-ocimene (5.7%)	[30]
	Serbia, flowers	octyl butanoate (26.1–29.7%), *γ*-palmitolactone (13.9–24.0%), hexyl butanoate (1.8–12.7%), octyl acetate (4.2–8.3%), *cis*-*β*-ocimene (3.2–6.6%), (*E*)-*β*-farnesene (3.4–6.0%), myristicin (3.8–4.0%), caryophyllene oxide (0.3–3.2%)	[22]
	Serbia, fruits	octyl butanoate (53.6–65.1%), octyl acetate (1.1–28.9%), *n*-octyl hexanoate (4.6–15.4%) hexyl butanoate (3.1–10.6%), *n*-octanol (1.0–3.5%)	[22]
	Serbia, leaves	*γ*-palmitolactone (22.6–29.5%), (*E*)-*β*-farnesene (4.4–13.8%), caryophyllene oxide (8.0–10.6%), (*E*)-caryophyllene (8.2–9.9%), (*Z*)-*β*-ocimene (3.2–7.4%), octyl butanoate (2.0–7.1%), (*E*)-*β*-ocimene (4.1–6.3%), germacrene D (3.4–4.6%), *β*-bourbonene (1.7–3.1%),	[22]
	Serbia, stems	*γ*-palmitolactone (50.6–53.4%), (*E*)-*β*-farnesene (4.9–6.5%), caryophyllene oxide (2.1–6.1%), (*Z*)-*β*-ocimene (3.2–5.5%), (*E*)-nerolidol (0–4.5%), myristicin (1.1–3.8%), hexadecanoic acid (2.3–3.3%)	[22]
	Serbia, roots	myristicin (39.7–62.1%), terpinolene (1.7–23.4%), (*Z*)-falcarinol (10.5–15.9%), *γ*-palmitolactone (1.5–15.6%)	[22]

**Table 3:** Minimum Inhibitory Concentrations (MICs) against several bacteria species.

Strains	MIC [µg/mL] ± SD	*p*-Value
***E. coli***	24,333.3 ± 1527.5	**
***P. aeruginosa***	25,000 ± 1732.1	****
***S. typhimurium***	>25,000 ± 0	****
***B. subtilis***	22,666.7 ± 2516.6	***
***B. cereus***	18,333.3 ± 2886.8	****
***M. smegmatis***	15,000 ± 5000	****

MICs were determined by the broth microdilution method against Gram-positive and Gram-negative strains. Values represent the mean of three independent experiments. MIC values are expressed in µg/mL, and results are presented as mean ± standard deviation (SD), with corresponding *p*-values. Statistical significance was assessed by two-tailed paired *t*-test relative to untreated cells. Significance is indicated as follows: *p* ≤ 0.01 (**), *p* ≤ 0.001 (***), *p* ≤ 0.0001 (****).

## Data Availability

All data and materials are available upon request from the corresponding author.

## Supplementary Materials

The following supporting information can be downloaded at: https://www.mdpi.com/article/10.3390/molecules30143033/s1, Figure S1: Population of *Pastinaca sativa* subsp. *sativa* collected in San Candido, Bolzano, South Tyrol (Italy).
