# Supplementary material for: South Tyrol (Italy) Pastinaca sativa L. subsp. sativa Essential Oil: GC-MS Composition, Antimicrobial, Anti-Biofilm, and Antioxidant Properties
Source: Molecules. 2025 Jul 19;30(14):3033. doi: 10.3390/molecules30143033 (PMC12298367; doi:10.3390/molecules30143033)
Supplement: Supplementary file 1 [file molecules-30-03033-s001.zip › molecules-3686439-supplementary.pdf]

Supplementary Materials

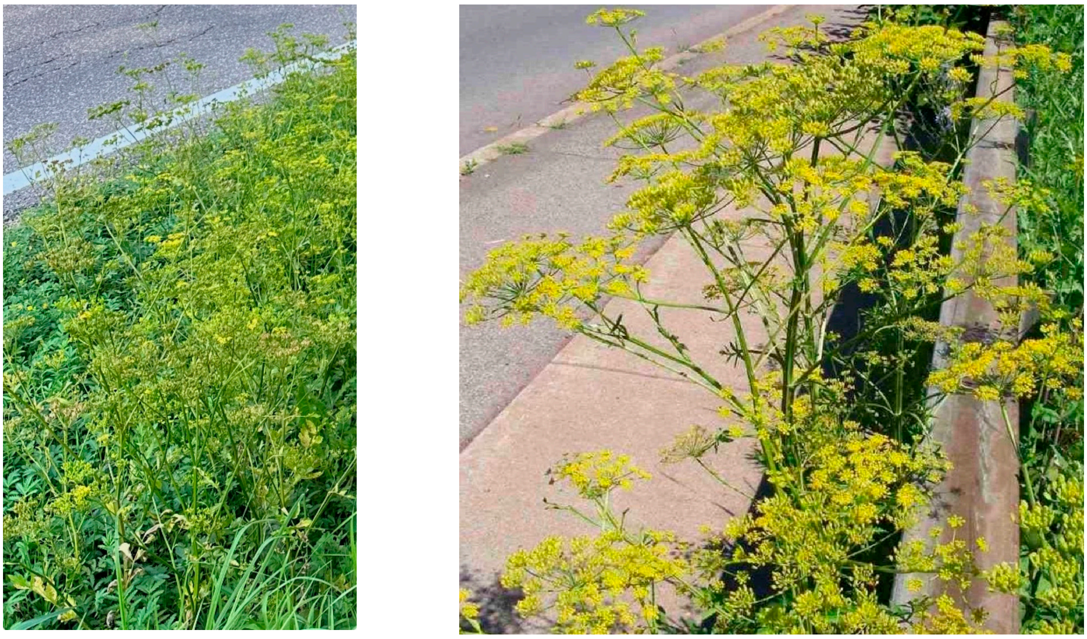

Figure S1. Population of *Pastinaca sativa* subsp. *sativa* collected in San Candido, Bolzano, South Tyrol (Italy).
